# Holistic Performance Programming for mTBI Recovery in U.S. Military Tactical Athletes: A Narrative Review

**DOI:** 10.3390/sports14050195

**Published:** 2026-05-09

**Authors:** Ed Daly, John Mackersie, Lisa Ryan

**Affiliations:** 1 Department of Sport, Exercise & Nutrition, School of Health, Sport Science & Nutrition, Atlantic Technological University, H91 T8NW Galway City, Ireland; ed.daly@atu.ie; 2 United States Special Operations Command (USSOCOM), Tampa, FL 33621, USA; john.c.mackersie.ctr@socom.mil

**Keywords:** military, tactical athletes, mTBI, recovery, holistic programming

## Abstract

Tactical athletes, including military service members, are exposed to occupational demands that increase their risk of mild traumatic brain injury (mTBI), particularly through blast exposure, falls, collisions, and repeated sub-concussive events. Although clinical tools and progressive return-to-activity protocols support acute management, recovery may remain fragmented when physical, cognitive, psychological, and performance domains are not integrated. Military personnel require recovery models which extend beyond symptom resolution and return-to-duty clearance. Holistic performance programming offers a multidimensional framework which incorporates subject matter experts across strength and conditioning, rehabilitation, nutrition, behavioural health, cognitive performance, and human performance optimisation. This narrative review examines the role of holistic performance programming in optimising recovery from mTBI among tactical athletes, with emphasis on interdisciplinary care, structured assessment, recovery periodisation, monitoring technologies, and return-to-duty readiness. The role of embedded subject matter experts in identifying and monitoring mTBI; interdisciplinary care models which integrate clinical and performance expertise; structured recovery pathways from assessment to reintegration; and the importance of flexibility, communication, and service member engagement are examined. In addition, the review assesses the potential use of biomarkers, wearable technologies, and multi-domain assessment tools to guide individualised recovery. Holistic performance programming may bridge the gap between clinical recovery and operational readiness following mTBI. By integrating physical, cognitive, psychological, nutritional, and sleep-related strategies, this approach may reduce fragmented care and better address the complex nature of mTBI recovery. Interdisciplinary performance teams may improve early recognition, individualised rehabilitation, safer return-to-duty decisions, and long-term readiness. Future practice should prioritise standardised assessment, real-time monitoring, education, and stigma reduction.

## 1. Introduction

### Understanding TBIs in the Tactical Environment

Mild traumatic brain injury (mTBI) remains a substantial concern within military populations, with implications for both short- and long-term cognitive, emotional, and physical functioning. They are a common occupational injury in military settings. mTBIs may occur through various mechanisms, with blast exposure being a primary contributor [1,2]. Explosive blasts produce rapid overpressure followed by a vacuum effect, causing both direct and secondary brain trauma [3,4]. These effects are often compounded by physical impacts with the environment such as falls or accidental collisions with equipment [5]. Close-quarter combat, breaching operations, and extended weapon training may expose service members to repeated sub-concussive forces. These may not result in immediate symptoms but may accumulate in some cases to cause long-term neurological consequences [6]. Although the acute clinical management of mTBI is well established, there is a growing recognition of the need for integrative strategies which address the multifaceted demands of military service.

The Department of Defence (DoD) and Traumatic Brain Injury Centre of Excellence (TBICoE) continue to report on large volumes of TBIs with mTBIs, or concussions, making up the majority of cases [7,8]. As a consequence, there are varied levels of return to active service depending on the severity and timing of the injury [9]. In many cases of mTBI, the signs and symptomology can oftentimes present with insidious or imperceptible alterations to overall cognitive function, or present with alterations to emotional states which may be overlooked or misattributed [10]. However, it is now understood that the cumulative effects of these mTBIs can result in chronic impairments which may affect memory, balance, attention, and decision-making [11]. In addition to these impairment factors, the cumulative effect of multiple mTBIs may increase susceptibility to an increase in symptom severity and elongate recovery times [12].

Current research has demonstrated that even low-level blast exposure can induce neuroinflammation and structural changes which may be detectable via neuroimaging [13]. As mTBI incidence continues to be a considerable issue for military personnel, it is imperative that military institutions are more proactive in their approach to screening, documentation, and interventions which aim to mitigate against long-term disability and enhance service member readiness [14,15,16].

Currently, the progressive return-to-activity (PRA) protocol represents a structured, symptom-guided process designed to facilitate the safe and effective reintegration of service members following an mTBI. The process outlines a staged approach which gradually increases physical and cognitive demands, which ensures progression occurs when symptoms have resolved or stabilised at each phase. This framework, which ranges from rest and light activity to unrestricted duty, aims to minimise the risk of symptom exacerbation and secondary injury, while promoting functional recovery and operational readiness [17]. Importantly, the PRA emphasises individualised care by accounting for a service member’s history of prior TBI, recognising that cumulative injuries may influence recovery timelines and tolerance to activity progression.

Completion of the Military Acute Concussion Evaluation 2 (MACE 2) is mandated for all populations exposed to potentially concussive events; this process ensures standardised screening and documentation. MACE 2 provides a structured assessment of cognitive, neurological, and symptom domains which support early identification and management of mTBI. In addition, the TBICoE provides an extensive suite of clinical practice guidelines (CPGs) and recommendations addressing TBI-related sequelae such as sleep disturbances, cognitive impairments, and mood alterations facilitating consistent, evidence-based care across military and clinical settings [8].

While standardised assessments and clinical guidelines establish a medical foundation for the management of mTBI, they often focus on symptom resolution and return to baseline functioning rather than long-term performance optimisation. To assist in the recovery process, a more comprehensive, holistic framework which integrates physical, cognitive, and psychological rehabilitation is needed to support sustained recovery and enhance readiness among service members.

Holistic performance programming offers an integrated and adaptive framework which may be useful in mTBI rehabilitation instead of, or in tandem with, traditional symptom-guided management. Unlike standard approaches which focus mainly on acute symptom resolution, holistic performance programming models incorporate strength and conditioning (S&C), cognitive retraining, psychological resilience, nutritional support, and sleep optimisation as interconnected domains of recovery [18]. This multidimensional approach facilitates both neural and physical restoration while enhancing adaptability, resilience, and operational readiness [18].

Further research related to holistic programming for service members with co-occurring post-traumatic stress disorder (PTSD) is warranted. As PTSD and mTBI may frequently overlap, integrating psychological, physical, and cognitive rehabilitation within a holistic framework may improve emotional regulation, reduce symptom persistence, and promote sustained performance outcomes [19,20].

This approach may present a promising avenue for optimising both recovery and sustained performance in active service members post-injury. A comprehensive review of holistic performance programming in military settings is necessary to define best practices, identify evidence gaps, and support the development of personalised, mission-ready rehabilitation pathways.

Notably, the existing literature provides guidance on the acute clinical management of mTBI; much of this evidence remains organised around individual domains of care, such as symptom monitoring, neurocognitive assessment, physical rehabilitation, behavioural health, sleep, nutrition, or return-to-activity progression [18,19]. What remains less clearly developed is an interdisciplinary synthesis which explains how these domains can be coordinated within a practical human performance framework for tactical athletes [20]. In particular, limited guidance exists on how clinical rehabilitation, strength and conditioning, cognitive recovery, psychological support, nutrition, sleep optimisation, and readiness monitoring can be integrated to support both recovery and sustained operational performance.

This review aims to synthesise the clinical, rehabilitation, and human performance literature into a holistic performance programming model for U.S. military tactical athletes recovering from mTBI. Rather than focusing solely on symptom resolution or return-to-duty clearance, this review positions mTBI recovery as a multi-domain and iterative process requiring coordinated input from interdisciplinary subject matter experts. By bridging recovery and performance optimisation, holistic performing programming approaches may enhance return-to-duty readiness, reduce long-term disability, and improve quality of life.

Table 1 provides a conceptual comparison between traditional symptom-guided mTBI care and holistic performance programming. It was developed from themes identified in the reviewed literature and organised around five criteria: focus of care, assessment approach, professional roles, adaptability of intervention, and intended outcomes.

The table shows how holistic performance programming may complement established clinical guidance by extending care beyond acute symptom management toward integrated physical, cognitive, psychological, and performance-based recovery.

## 2. Methods

A narrative literature review was conducted to examine the role of holistic performance programming in supporting recovery, rehabilitation, and return-to-duty readiness among tactical athletes following mild traumatic brain injury (mTBI). The review focused on the literature relating to military service members, tactical athletes, and included areas such as mTBI, post-concussive symptoms, interdisciplinary rehabilitation, performance programming, strength and conditioning, cognitive recovery, nutrition, sleep and return-to-duty frameworks.

A structured literature search was undertaken across electronic databases such as CINAHL, PubMed, Science Direct, and Web of Science. The search was limited to articles published between 2020 and 2025 to ensure relevance to contemporary military, clinical, and performance-based approaches to mTBI management. Search terms included combinations of keywords and phrases such as “mild traumatic brain injury,” “mTBI,” “concussion,” “military,” “service members,” “tactical athletes,” “holistic performance,” “human performance,” “return to duty,” “rehabilitation,” “post-concussion symptoms,” “interdisciplinary care,” “strength and conditioning,” “cognitive rehabilitation,” “wearable technology,” and “biomarkers.”

Records were excluded if they were not focused on mTBI or concussion, were not relevant to military or tactical populations, did not address rehabilitation, recovery, performance programming, or return-to-duty considerations, or were outside the conceptual scope of holistic performance programming.

Data from the included records were synthesised narratively rather than statistically. Key findings were organised thematically according to the major domains of holistic performance programming identified in the literature, including interdisciplinary care models, the role of subject matter experts, structured recovery and reintegration, cognitive and psychological support, strength and conditioning, monitoring technologies, biomarkers, and future directions for military mTBI care. Given the narrative design of the review, no meta-analysis was conducted. Instead, the synthesis aimed to integrate current evidence, identify gaps in practice and research, and provide a conceptual overview of how holistic performance programming may support recovery and sustained readiness in tactical athletes following mTBI.

## 3. Holistic Performance Programming in Military Settings

### 3.1. The Role of Subject Matter Experts (SMEs) in mTBI Management

The integration of specialist performance professionals or subject matter experts (SMEs) into military units may enhance the early identification and management of occupational injuries in military settings. These professionals possess a wide range of proficiencies which include expertise in athletic training, physical therapy, strength and conditioning coaching, and cognitive specialists. SMEs are frequently embedded within units and can function as the first line of observation and support for holistic performance programming due to the close proximity of contact with military service members [21,22].

Due to these regular contacts, SMEs are ideally placed to identify and manage occurrences of mTBI. Screening tools such as the MACE 2, Balance Error Scoring System (BESS), and reaction-time assessments may be used to monitor any early signs of neurological dysfunction [23]. These close social support interactions may allow SMEs or performance professionals to identify behavioural changes such as irritability, slowed reactions, or confusion which may be unobserved in standard clinical evaluations [24,25].

Furthermore, human performance specialists may play a role in reducing stigma by influencing the environmental culture [26]. By fostering trust and rapport, they create an environment where service members feel safe disclosing symptoms without fear of career repercussions [25]. Programmes such as TBICoE offer evidence-based protocols and training that equip performance teams to operate confidently within a multidisciplinary model [27,28].

### 3.2. Interdisciplinary Holistic Performance Programming in the Military

Since the mid-2000s, holistic performance programmes have emerged within both special operations and conventional forces [29]. Notable examples include the U.S. Army’s Holistic Health and Fitness (H2F) programme and Special Operations Command’s Preservation of the Force and Family (POTFF) initiative [30,31]. These models emphasise the integration of physical, psychological, cognitive, and spiritual domains to optimise total force readiness [32].

SMEs are central to the development and implementation of clinical assessment tools and suggesting clinical recommendations in practical settings [33]. The resulting recommendations can be managed by an interdisciplinary team within these programmes, which typically consist of physical therapists, S&C coaches, nutritionists, dietitians, behavioural health providers, and medical practitioners [34]. This integrated collaborative approach ensures that performance and rehabilitation programmes are customised, data-driven, and synchronised with unit operational needs [35].

There is a growing body of research to support the efficacy of these types of interdisciplinary approaches. Integrative intensive outpatient programmes (IOPs) are structured, short-term, interdisciplinary rehabilitation models for service members and veterans with persistent post-concussive symptoms (PPCS) often compounded by PTSD, depression, and anxiety. They deliver integrated physical, cognitive, and behavioural care without requiring inpatient admission. In many instances, participants in IOPs demonstrate measurable improvements in both cognitive function and emotional well-being. For instance, De Graba et al. [36] reported that patients treated in IOP settings displayed meaningful improvements across various outcome measures such as generalised anxiety and satisfaction with life scales. Their study [36] reported significant, clinically meaningful improvements on all measures at discharge with the largest effects related to anxiety and depression, and group-level benefits were sustained for up to six months. This programme was carried out over a four-week duration and had components such as interdisciplinary neuro-rehab, creative arts and family education programmes. However, the study had limitations such as the absence of a control group and the use of self-reported outcome measures. The effectiveness of the programme may have been affected by the multimodal aspects of care [36].

Other research related to the implementation of IOPs, such as Home Base, NICoE and Veteran Affairs (VA) reported that the period of intervention varied, with some of the most common being two weeks in duration [37]. These shorter support programs, for example Home Base, tend to emphasise massed psychotherapy plus targeted cognitive rehab, whereas the NICoE programme is longer and more integrative which combines components such as neuro-rehab, behavioural health, headache care, sleep and complementary therapies [36,38]. The Department of Veterans Affairs (VA) deliver mTBI care through the nationwide Polytrauma/TBI System of Care, guided by the 2021 VA/DoD mTBI clinical practice guidelines which recommend a symptom-focused, interdisciplinary management structure with the treatment of comorbidities [39].

Home Base and NICoE report meaningful short-term symptom improvements after time-limited intensive care [20]. The current evidence from VA emphasises guideline-concordant, symptom-targeted care and long-term outcomes from large longitudinal cohorts; randomised trials for veterans with mTBI and PTSD may refine the cognitive rehabilitation efficacy of this approach [40]. In general, the design of IOPs are uniformly observational, and it is not clear whether any existing programmes provide randomised comparisons to standard care; therefore, causal inference is limited. In addition, the outcome focus may differ. For example, NICoE reports broad symptom relief sustained for six months, where other programmes increasingly track participation and role functioning or examine symptom subgroups to personalised care [41]. These programmes are generally provided at no cost to active military personnel or veterans; however, there are issues with the generalisability and scalability of IOPs as the majority of these programmes run in specialised centres with substantial interdisciplinary resource-intense requirements [20]. Across IOP models, common elements include interdisciplinary assessment, symptom education, behavioural health support, cognitive and physical rehabilitation, and individualised goal setting. These shared components suggest that multimodal care is particularly relevant for service members with persistent post-concussive symptoms, especially when mTBI co-occurs with PTSD, anxiety, depression, sleep disturbance, chronic pain, or reduced occupational participation.

However, programmes differ in duration, intensity, scope, and setting. Home Base and similar models often emphasise massed psychotherapy and targeted cognitive rehabilitation, whereas NICoE-type programmes provide broader interdisciplinary care, including neurological evaluation, headache management, sleep support, complementary therapies, family education, and functional rehabilitation [20]. VA-based services are typically embedded within longer-term care systems and emphasise symptom-targeted rehabilitation, comorbidity management, continuity of care, and reintegration into daily function [37].

These differences affect scalability, cost, staffing, patient selection, and interpretation of outcomes. Although IOPs show promise, variation in programme design limits direct comparison. Future studies should clarify which components are most effective, for whom, and under which operational conditions. Overall, IOPs have demonstrated their effectiveness for improved outcomes and higher occupational reintegration rates among service members [42].

### 3.3. Structured Recovery: From Assessment to Reintegration

As previously stated, the management and recovery from mTBI or persistent post-concussion symptoms may be a convoluted process [43,44]. This process may be enhanced by the integration of multimodal treatment options due to the variety and nature of mTBI symptoms [45]. Symptom progression can vary widely based on many factors such as mTBI severity, history of previous documented mTBIs and the cumulative effects of sub-concussive incidence [46]. In the absence of a structured and integrated treatment programme, the overall recovery pathway for service members can become fragmented or may fail to address key domains of recovery [47,48]. Common barriers include limited resources and infrastructure, especially in austere or deprived environments where access to advanced technology, stable power, or connectivity is constrained [7]. Organisational, associated costs and cultural resistance, along with inconsistent leadership support, can further hinder implementation [7].

Generally, a structured recovery process begins with a multi-domain assessment, which encompasses clinical evaluations, cognitive performance metrics, functional movement screening, athletic status assessments and psychological inventories. For example, based on the results from these assessments, the S&C SME will be enabled to construct a comprehensive and individualised physical conditioning plan, modelled on established periodisation principles [49]. These principles are based on the fundamental principles of training which aim to balance the frequency, intensity, time and type of workload which assists in recovery [50].

In this interdisciplinary model, the early stages focus on symptom stabilisation and clinical treatment. As symptoms begin to subside, the emphasis shifts toward S&C, cognitive stimulation, and psychological readiness [51]. Reintegration to active service may include simulated duty tasks, progressive return-to-training protocols, and team-based exercises to restore confidence and rebuild functional endurance.

In many individual situations, cognitive function can be accepted as a notable predictor of return-to-duty success. A study by Lesniak et al. [52] found that using objective measures of neuropsychological scores correlated with predictors of successful reintegration for military populations. These findings highlight the critical role of SMEs in monitoring cognitive recovery alongside physical rehabilitation.

### 3.4. Flexibility and Communication

As previously discussed, in many cases, the recovery process related to mTBI may have unpredictable aspects and can frequently be non-linear in nature. In many instances, recovery can be influenced by the presentation of a complex range of symptomologies and co-occurring bodily injuries. These factors can necessitate a degree of flexibility in the overall treatment plan [53]. Persistent post-concussion symptoms (PPCS) such as fatigue, dizziness, irritability, headaches and concentration issues may resurface during stress or exertion, which may require immediate adjustments for SMEs [54]. In these situations, it is integral that the interdisciplinary team meet regularly and discuss the reassessment options available to progress and modify treatment plans in real time.

Communication between SMEs and the service member is paramount. When care plans are collaborative and transparent, engagement improves and setbacks are addressed more constructively. SMEs must validate the invisible nature of brain injury and provide social support for emotional recovery, especially in high-performing populations unaccustomed to vulnerability [55,56].

### 3.5. Monitoring S&C Progress and Sustaining Rehab Training Engagement

As technological advances in human health monitoring progress in an ever-increasingly rapid manner, it is crucial for interdisciplinary teams to leverage these advanced technologies to enhance the holistic care of active service members [57]. There are a multitude of monitoring strategies which range from subjective reporting to objective biomarkers. For example, self-report tools such as the Neurobehavioral Symptom Inventory (NSI) or Headache Impact Test (HIT-6) provide insight into daily challenges [58]. Technological innovations such as wearable sensors and mobile applications are being explored to enhance real-time monitoring. For instance, heart rate variability (HRV) tracking may help detect autonomic nervous system disruption, while eye-tracking and electroencephalogram (EEG)-based devices can assess neurological status in field environments [59].

HRV and EEG are increasingly being studied as physiological indicators of autonomic and cortical recovery following mTBI. Increased HRV post-intervention may reflect an enhanced parasympathetic regulation [60]. EEG changes, such as the restoration of event-related potentials, may indicate reorganisation of cortical networks and improved cognitive processing [61]. However, findings remain inconsistent across studies, and the precise relationship between HRV or EEG modulation and functional recovery after TBI remains incompletely defined [60,61]. In conjunction with these advanced technical monitoring methods, human performance specialists can include a variety of functional assessments which can evaluate mobility, test endurance, and the overall cognitive load capacity of the service member.

**Figure 1:**
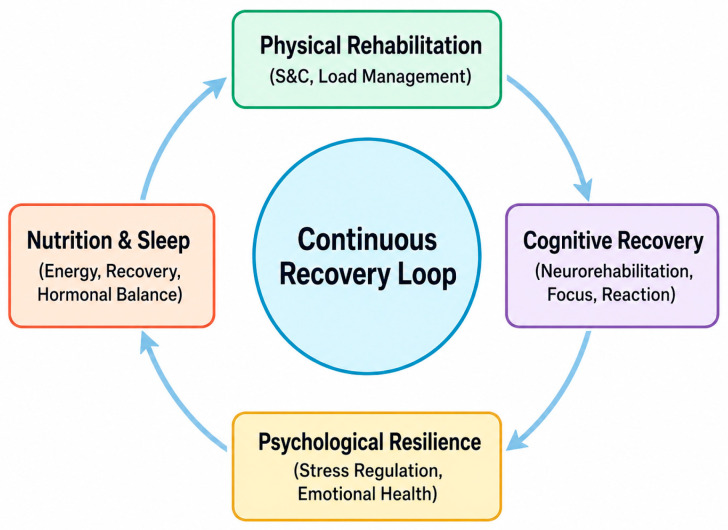
Circular system model for holistic performance recovery.

The primary objective for all members of the interdisciplinary team is to sustain engagement throughout the recovery process. When service members are educated on the links between the recovery process, the acknowledgment of symptomology and their long-term performance, it may lead to a deeper understanding of the rationale behind each intervention [62]. In turn, this may lead to service members being able to visualise their personal progress and they may be more likely to adhere to treatment plans and report setbacks early. Programmes which reinforce a long-term view of health beyond return to duty contribute to sustainable well-being and post-service quality of life. For example, Figure 1 represents a model of dynamic and interdependent holistic recovery, with an emphasis on feedback and adaptation across multidisciplinary domains to sustain performance and readiness after mTBI.

## 4. Discussion and Future Directions

While integrated performance programming demonstrates promise to military mTBI care, challenges remain and continued advancements are required. Although the reviewed literature supports interdisciplinary and holistic approaches, the overall quality of evidence remains variable. Much of the evidence comes from observational studies, retrospective cohorts, programme evaluations, narrative reviews, and scoping reviews, with limited randomised controlled trials. Many studies also include mixed populations of active duty personnel, veterans, athletes, and civilians, which limits generalisability to currently serving U.S. military tactical athletes.

Methodological limitations include reliance on self-reported symptoms, small or selective samples, lack of control groups, variable follow-up periods, and inconsistent outcome measures. Important factors such as prior mTBI history, blast exposure, PTSD, sleep disturbance, pain, musculoskeletal injury, and redeployment pressures are not always consistently measured or controlled, despite their relevance to recovery and readiness.

Despite significant progress in acute-phase interventions, the complexity of mTBI presentation and recovery remains a challenge. These challenges are particularly notable for those who are regularly exposed to repetitive blast injuries, unpredictable redeployment or veterans [63]. In addition, research into personalised treatment strategies would be beneficial, as recovery rates vary widely depending on psychosocial and environmental factors [18]. There are several key areas of study which would support the further development of evidence-based return-to-duty protocols and performance sustainability frameworks.

### 4.1. Longitudinal Studies Examining the Trajectory of Recovery Across Occupational Specialties and Service Branches

There are longitudinal studies which examine the trajectory of recovery from concussion and mTBI in military populations which provide further insights into the variability of outcomes across occupational specialties and service branches. These types of studies may track individuals over extended periods which may allow researchers to study the non-linear nature of recovery and the interplay of biological, psychological, and environmental factors unique to military settings [64].

For example, the Long-Term Impact of Military-Relevant Brain Injury Consortium Chronic Effects of Neurotrauma Consortium (LIMBIC-CENC) Prospective Longitudinal Study (PLS) tracks previously deployed service members and veterans with and without history of combat-related mTBI and monitors them over time to detect trajectories of cognitive, functional, biomarker, imaging, and symptom change [65]. In addition to self-reporting measures, functional, quality of life and degeneration metrics are incorporated [66]. The National Collegiate Athletic Association (NCAA) DoD Concussion Assessment, Research and Education (CARE) Consortium, is an ongoing prospective study tracking the ‘natural history’ of concussion which includes U.S. Military Service Academy cadets in conjunction with NCAA athletes [67]. This longitudinal study repeatedly assesses symptoms, neurocognitive performance, balance, and return to activity to characterise recovery trajectories. In addition, the consortium includes a long-term brain health phase for service members and athletes [68].

The Millennium Cohort Study (MCS) is a long-running cohort study which repeatedly surveys and tracks service members and veterans. Recent work examines blast exposure and subsequent mTBI diagnoses and other outcomes, enabling the production of multi-year trajectories in post-service populations [69]. The Armed Services Trauma and Rehabilitation Outcome TBI (ADVANCE-TBI), which involves the Armed Forces of the United Kingdom, is an extension of the twenty-year ADVANCE cohort of the UK Afghanistan-deployed military personnel. It utilises mTBI phenotyping methods such as biomarkers and genetics, with repeated follow-ups to investigate long-term neurological and psychosocial outcomes after mTBI [70]. Additional longitudinal studies include the VA TBI Model Systems (TBIMS) and the VA/DoD observational studies.

The TBIMS aims to understand and improve the rehabilitation outcomes for veterans. TBIMS is a multicentre longitudinal programme following individuals with TBI after comprehensive inpatient rehabilitation. This cohort includes veterans and active duty service members, and characterises the recovery trajectories over time [71]. The VA/DoD observational cohort study analyses multi-year trajectories with research into mTBI, PTSD and disability. In addition, there may be differences within the various service branches which may influence recovery outcomes. For example, variations in training, deployment cycles, access to care for simple or complex mTBI, and post-injury rehabilitation protocols may influence recovery timelines [72]. These types of studies may contribute to refining return-to-duty guidelines by identifying potential biomarkers, neurocognitive trends, and psychosocial factors predictive of long-term outcomes. This may support more individualised, occupation-sensitive care pathways and policy decisions aimed at optimising brain health in military service members.

These longitudinal studies are relevant to the proposed holistic model because they address mTBI recovery as a dynamic, non-linear process. Holistic performance programming requires repeated assessment across clinical, cognitive, psychological, physical, occupational, and physiological domains to identify delayed recovery, persistent symptoms, readiness changes, and risk factors that may not be evident during acute assessment.

Future longitudinal research should test holistic performance programming as an integrated model rather than examining isolated outcomes. Such designs may help determine whether coordinated interdisciplinary care improves long-term function, reduces symptom recurrence, and enhances readiness compared with standard symptom-guided approaches.

### 4.2. Standardisation of Multi-Domain Assessment Tools to Streamline Care

As mentioned previously, mTBI can present with a diverse range of symptoms which necessitates a comprehensive, multi-domain evaluation approach for SMEs. The standardisation of multi-domain assessment tools for mTBI may streamline care and enhance data comparability across military service branches [73]. Standardised tools could assist interdisciplinary teams in capturing comparable data across units and time points, fostering more coherent clinical pathways and improving interoperability within joint operations. This streamlining may require the integration of electronic health records and ongoing SME training to ensure accuracy of use in operational and recovery settings. Data may be pooled for large-scale analyses, accelerating the discovery of prognostic indicators and enabling the refinement of treatment guidelines. This is particularly important in identifying patterns of delayed recovery or comorbidities, which often co-occur with mTBI [74].

### 4.3. Using Biomarkers

Emerging research suggests that biomarkers (serum-based) hold promise for predicting recovery timelines following mTBI service members. These biomarkers offer objective, quantifiable measures of injury severity and physiological recovery. In turn, these biomarkers may potentially allow SMEs to tailor treatment and make more precise return-to-duty decisions than symptom-based assessments alone [75].

Serum-based biomarkers such as glial fibrillary acidic protein (GFAP) and neurofilament light chain (NfL) have demonstrated diagnostic and prognostic potential in both civilian and military cohorts. Elevated levels of these proteins shortly after injury have been associated with more prolonged symptom duration, suggesting they could serve as early indicators of delayed recovery [76]. The Food and Drug Administration (FDA) have approved blood biomarker assays which measure GFAP and ubiquitin C-terminal hydrolase-L1 (UCH-L1). These are currently approved to aid the assessment of mTBI in adults [77]. These assays are designed for use within 12–24 h of a suspected mTBI injury. These point-of-care platforms provide rapid, objective data and represent significant progress toward standardised, evidence-based triage of head injuries in operational environments where imaging access is limited. Their minimally invasive collection makes them suitable for use in austere or deprived settings, enabling near-real-time assessment [78]. For military populations where operational readiness is critical, biomarkers show promise in terms of optimising medical resource allocation to reduce premature return to duty and improve long-term neurological outcomes. The integration of biomarker data may allow for individualised risk profiling and stratified care models.

### 4.4. Wearable Technology to Support Remote Monitoring

The adoption of wearable technology for remote monitoring in austere environments may present a transformative opportunity for enhancing the care of mTBI. In remote or combat settings where access to continuous SME supervision is limited, wearables may provide real-time physiological and behavioural data, enabling early detection of complications and facilitating evidence-based decision-making.

Current wearable devices have the ability to capture a range of metrics relevant to mTBI, including HRV, sleep patterns, gait stability, cognitive performance, and possibly oculomotor function [79]. These data streams, when analysed appropriately, may help assist SMEs in identifying deviations from recovery baselines, offering remote insights into the individual’s functional status.

In military contexts, wearables allow for passive, non-disruptive monitoring which aligns with the need for mobility. Additionally, wearable technology supports personalised care by providing objective feedback to the service member and interdisciplinary team, which may enhance compliance with rehabilitation protocols, improve health literacy and possibly indicate the ‘go/no go’ status of a service member [80].

Challenges remain, including device durability, data integration, user adherence, and privacy concerns. However, as technology matures, the strategic incorporation of wearable systems into mTBI management protocols holds significant potential for improving outcomes and maintaining force readiness, particularly in geographically dispersed or resource-limited military settings. Enhanced education campaigns to mitigate stigma and promote early symptom disclosure are necessary.

### 4.5. Education, Reporting and Awareness

Enhanced education campaigns are vital to mitigating stigma and promoting early symptom disclosure of mTBI in military service members. Despite increased awareness of mTBI, many service members continue to underreport symptoms due to concerns about career impact, perceived weakness, or fear of being removed from duty. This reluctance can delay diagnosis and treatment, increasing the risk of prolonged recovery and long-term cognitive or psychological complications [81].

Education campaigns tailored to military culture can reshape perceptions of mTBI by framing early symptom disclosure as an act of strength and responsibility critical to both personal health and mission readiness. These campaigns should emphasise that mTBI is a common and treatable injury, not a sign of weakness, and that early intervention significantly improves outcomes [82]. By addressing myths, reducing fear of repercussions, and providing clear pathways to care, enhanced education campaigns foster a culture where mTBI symptoms are reported and treated promptly. This not only protects the health of individual service members but also strengthens overall operational effectiveness and military readiness.

## 5. Conclusions

This review highlights the need for continued advancement in the identification, treatment, and long-term management of mTBI within active service members. The complex and often subtle presentation of mTBI which ranges from cognitive impairments to emotional disturbances can pose diagnostic challenges and may result in chronic deficits that undermine operational readiness and long-term quality of life.

The integration of SMEs, such as strength and conditioning coaches and cognitive specialists, into military units is an innovative step toward early identification and intervention. These professionals, through continuous contact with service members, play a pivotal role in screening, monitoring, and de-stigmatising brain injuries in operational settings. Additionally, the structured, periodised recovery approach which starts from assessment and progressing through rehabilitation and reintegration is an essential component to restore both functional capacity and confidence in service members.

Longitudinal studies are needed to understand variability in recovery across service branches along with the standardisation of multi-domain assessment tools to improve care coherence and inter-branch data comparability. The integration of wearable technologies for real-time physiological and cognitive monitoring would be a valuable asset for SMEs in terms of remote, field-based management of mTBI. Cultural reframing of mTBI as a manageable, treatable injury as opposed to a career threat may enhance care-seeking behaviour and improve outcomes across the force.

## Figures and Tables

**Table 1:** Comparison of symptom-guided and holistic performance programming care models.

Symptom-Guided mTBI Care	Holistic Performance Programming
Focuses on acute symptom management and staged progressive return to activity (PRA)	Integrates long-term recovery with performance optimisation across physical, cognitive, and psychosocial domains
Relies on clinician-driven assessment tools (e.g., MACE 2, TBICoE guidelines)	Combines clinical assessment with functional movement, neurocognitive, and readiness metrics
Involves primarily medical and rehabilitation professionals	Includes SMEs from S&C, nutrition, behavioural health, and cognitive science fields
Often time-limited and reactive to symptoms	Ongoing, adaptive, and proactive with recovery periodization and data-driven monitoring
Emphasises return-to-duty clearance	Emphasises sustained readiness, resilience, robustness and long-term well-being

## Data Availability

No new data were created or analysed in this study. Data sharing is not applicable to this article.
